# Supplementary material for: PKM2 aggregation drives metabolism reprograming during aging process
Source: Nat Commun. 2024 Jul 9;15:5761. doi: 10.1038/s41467-024-50242-y (PMC11233639; doi:10.1038/s41467-024-50242-y)
Supplement: Supplementary file 1 — Supplementary Information [file 41467_2024_50242_MOESM1_ESM.pdf]

Supplementary Fig. S1 related to Fig. 1

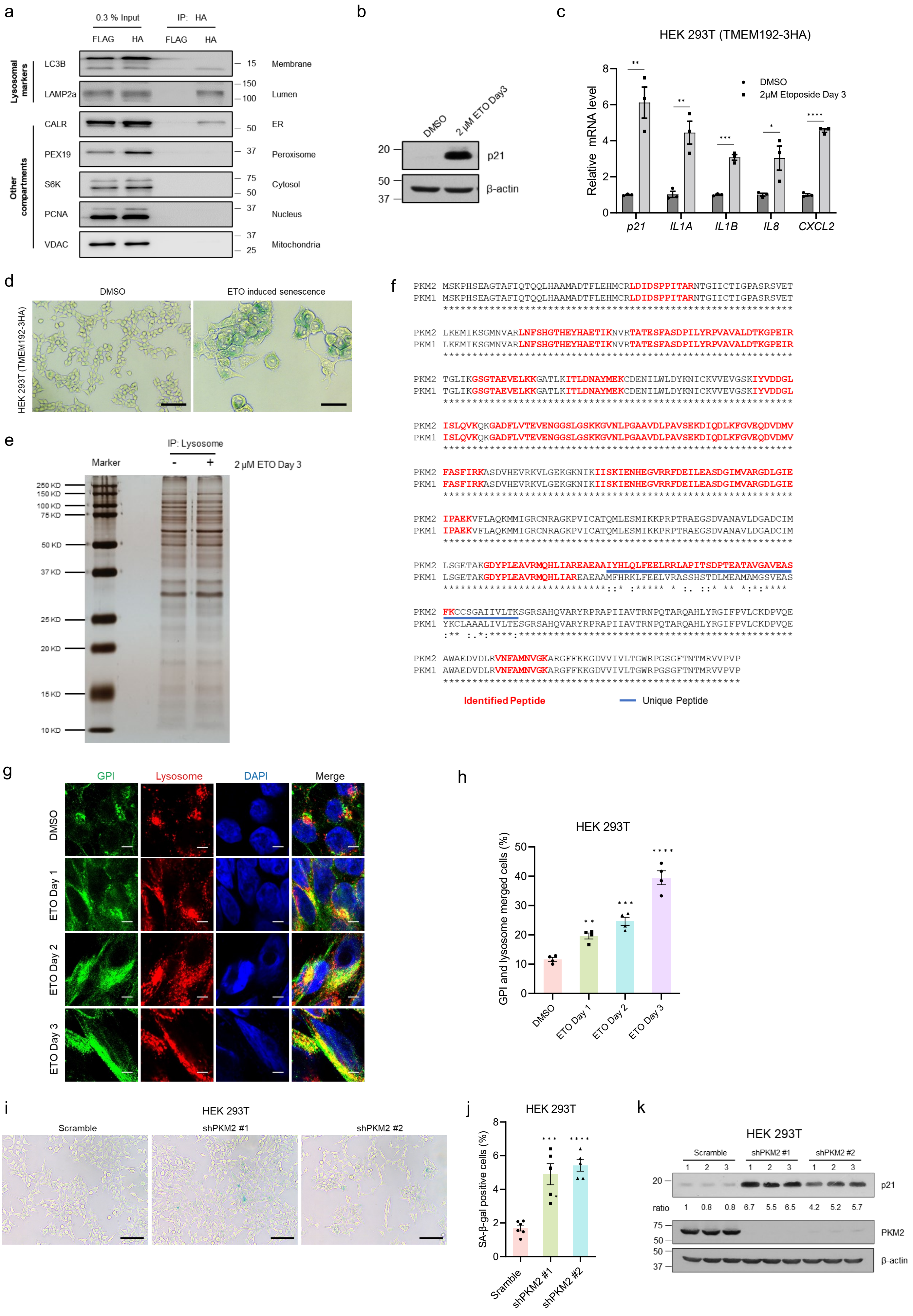

Supplementary Fig. 1 Glycolytic enzymes are enriched in lysosomes of senescent cells

a TMEM192-3HA or TMEM192-2Flag was stably expressed in HEK 293T cells which were subjected to lysosome immunoprecipitation using HA magnetic beads and subsequent Western blot analysis with different Verification of lysosome immunoprecipitation system by Western blot with different organelle markers. **b** HEK 293T (TMEM192-3HA) were treated with 2 μM etoposide (ETO) for three days followed by the detection of p21 protein level via Western blot. **c** HEK 293T (TMEM192-3HA) were treated with 2 μM etoposide (ETO) for three days followed by the detection of p21 and SASP mRNA level via Real-time PCR. n = 3, Two-tailed unpaired t-test was used. P = 0.0041 (*p21*), P = 0.0065 (*IL1A*), P = 0.0002 (*IL1B*), P = 0.0392 (*IL8*). **d** HEK 293T (TMEM192-3HA) were treated with 2 μM etoposide (ETO) for three days followed by SA-β-gal staining. Scale bar, 100 μm. **e** HEK 293T (TMEM192-3HA) were treated with 2 μM etoposide (ETO) for three days and then subjected to lysosome immunoprecipitation using HA magnetic beads and subsequent silver staining of the lysosomal proteins. **f** The alignment of PKM1 and PKM2 protein sequence. The peptides identified by MS in Fig. 1 are marked in red. The unique peptide PKM2 is marked with blue underline. **g**, **h** HEK 293T (TMEM192-3HA) were treated with 2 μM etoposide (ETO) for indicated days and then subjected to immunofluorescent imaging of GPI and lysosome (indicated by HA staining). Representative images were shown in (**g**). Scale bar, 5 μm. The percentage of GPI and lysosome merged cells was calculated in (**h**). n = 4, one-way ANOVA was used. P = 0.0074 (DMSO vs. ETO Day 1), P = 0.0002 (DMSO vs. ETO Day 2). **i**, **j** Scramble or PKM2 knock-down HEK 293T cells were subjected to SA-β-gal staining. Scale bar, 100 μm. P = 0.0001 (Scramble vs. shPKM2#1). **k** Scramble or PKM2 knock-down HEK 293T cells were subjected to detection of p21 protein level via western blot. *$P < 0.05$, **$P < 0.01$, ***$P < 0.001$, ****$P < 0.0001$. Error bars represent SEM. All the above experiments were repeated thrice on separate days with similar results. Source data are provided as a Source Data file.

Supplementary Fig. S2 related to Fig. 2

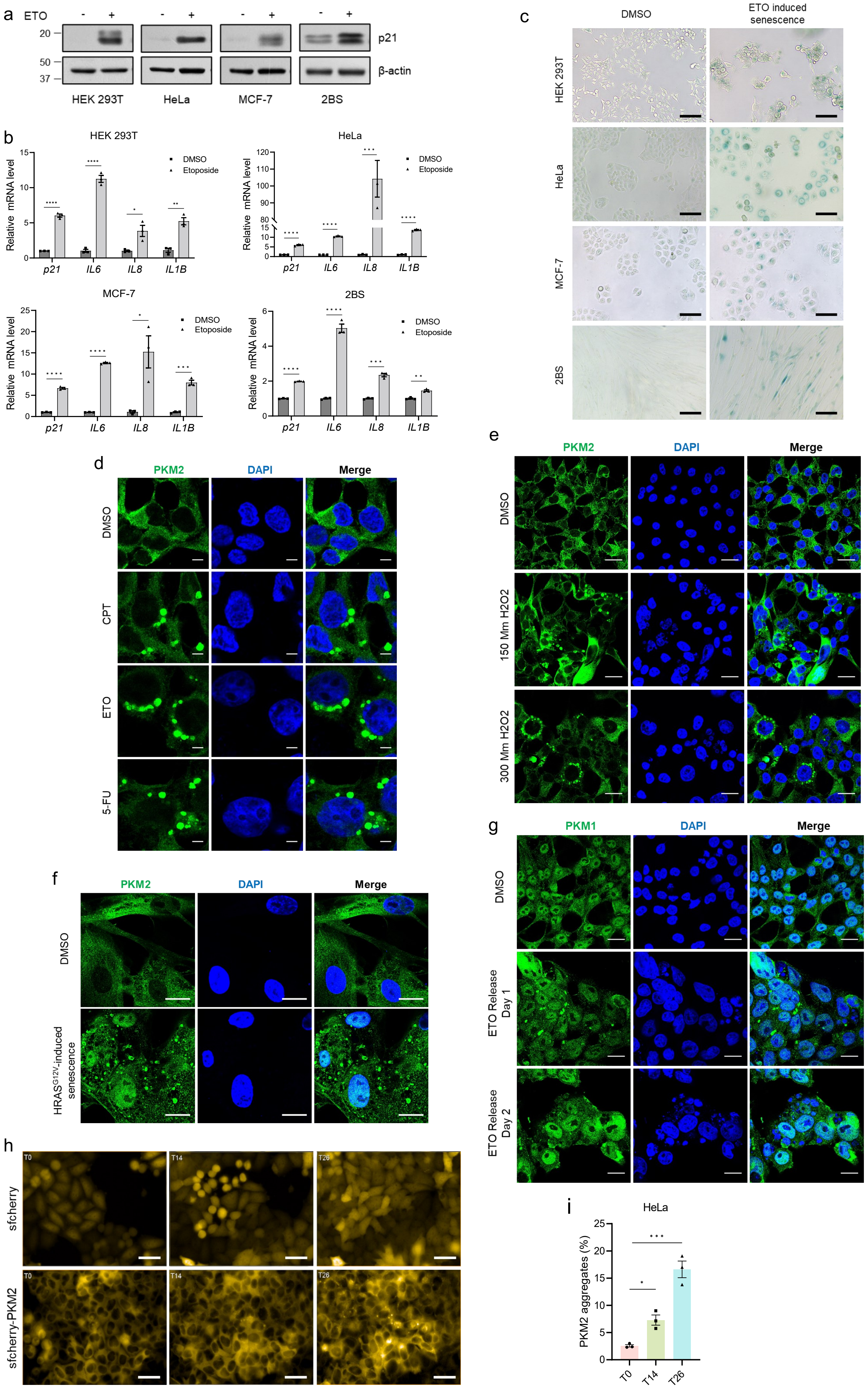

Supplementary Fig. 2 PKM2 tends to aggregate in senescent cells

a HEK 293T, HeLa, MCF-7, 2BS cells were treated with 2 μM etoposide for three days and subjected to detection of p21 protein level by Western blot. **b** HEK 293T, HeLa, MCF-7, 2BS cells were treated with 2 μM etoposide for three days and subjected to detection of p21 and SASP mRNA level by Real-time PCR. n = 3, Two-tailed unpaired t-test was used. $P = 0.0236$ (HEK 293T: *IL8*), $P = 0.0020$ (HEK 293T: *IL1B*); $P = 0.0007$ (HeLa: *IL8*); $P = 0.0202$ (MCF-7: *IL8*), $P = 0.0002$ (MCF-7: *IL1B*); $P = 0.002$ (2BS: *IL8*), $P = 0.0017$ (2BS: *IL1B*). **c** HEK 293T, HeLa, MCF-7, 2BS cells were treated with 2 μM etoposide for three days and subjected to SA-β-gal staining. Scale bar, 100 μm. **d** HEK 293T cells were treated with CPT (25 nM), etoposide (10 μM), 5-FU (100 mM) for 24 hours and release for two days followed by immunofluorescent imaging of PKM2. Scale bar, 5 μm. **e** HEK 293T cells were treated with H_2O_2 for 48 hours followed by immunofluorescent imaging of PKM2. Scale bar, 20 μm. **f** Scramble and $HRAS^{G12V}$-overexpressed fibroblasts 2BS were subjected to immunofluorescent imaging of PKM2. Scale bar, 20 μm. **g** HEK 293T cells were treated with etoposide (10 μM) for 24 hours and release for two days followed by immunofluorescent imaging of PKM1. Scale bar, 20 μm. **h**, **i** HeLa cells with sfcherry or sfcherry-PKM2 stably expressed were treated with 2 μM etoposide and subjected to immunofluorescent imaging dynamically using high-content immunofluorescent microscope. Representative images were shown in (**h**). Scale bar, 50 μm. The percentage of cells with PKM2 aggregates was calculated in (**i**). n = 3, one-way ANOVA was used. **i** $P = 0.0324$ (T0 vs. T14), $P = 0.0002$ (T0 vs. T26). $^{*}P < 0.05$, $^{**}P < 0.01$, $^{***}P < 0.001$, $^{****}P < 0.0001$. Error bars represent SEM. All the above experiments were repeated thrice on separate days with similar results. Source data are provided as a Source Data file.

Supplementary Fig. S3 related to Fig. 2

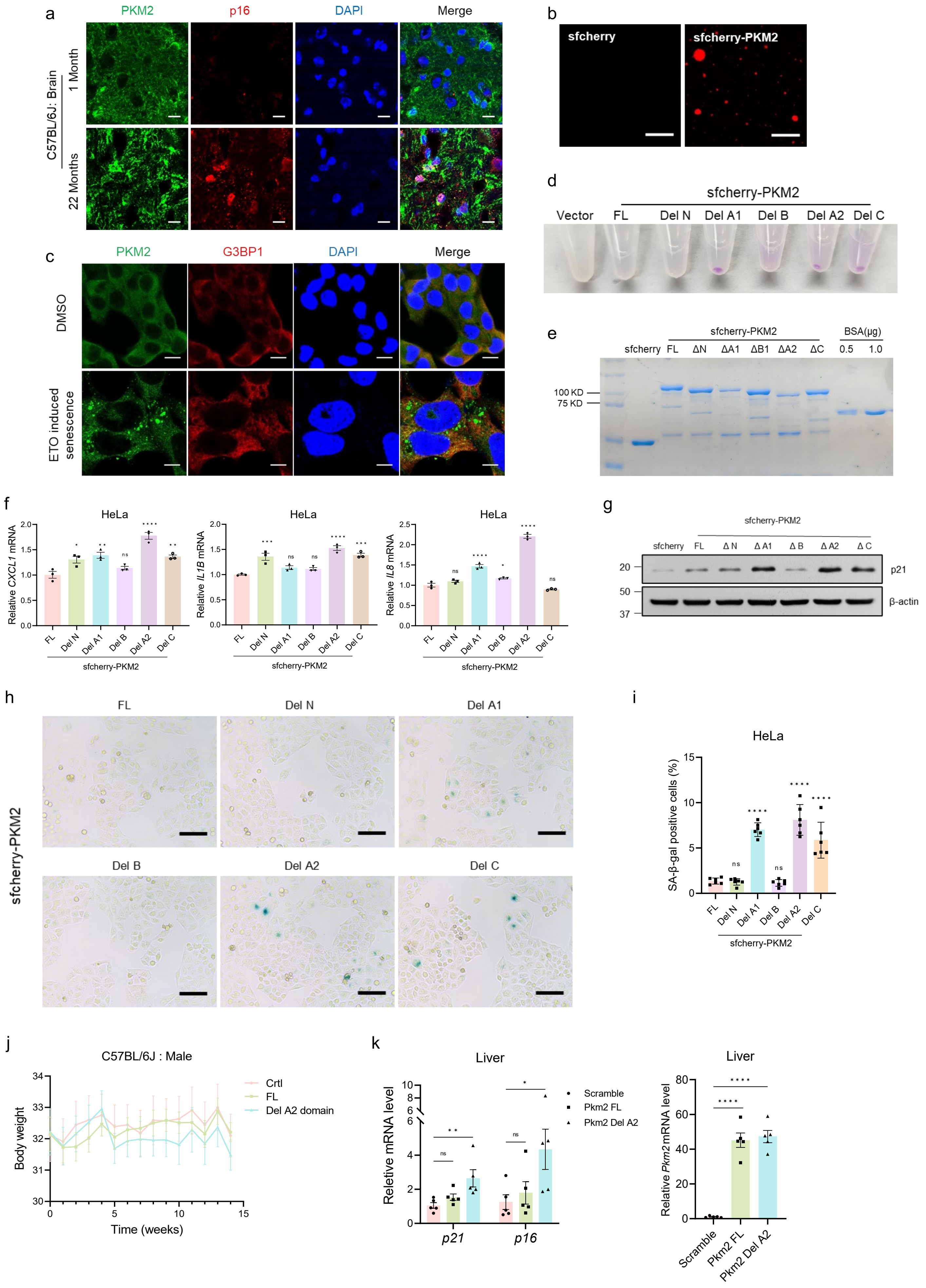

Supplementary Fig. 3 PKM2 aggregates involve in cellular senescence

a Brain of young and aged mice was prepared as frozen section for immunofluorescent imaging of PKM2. Scale bar, 20 μm. **b** sfcherry-PKM2 was purified and observed with confocal microscopy *in vitro*. Scale bar, 10 μm. **c** Senescent HEK 293T cells were induced by 2 μM etoposide (ETO) and subjected to immunofluorescent imaging of PKM2 and G3BP1 (stress granule marker). Scale bar, 10 μm. **d** HEK 293T cells were transfected with full length or truncated variants with deletion of any domain of sfcherry-PKM2 and lysed with lysis buffer for 40 minutes followed by centrifuge at 1,000 x g for 5 minutes at 4°C. **e** Full length or truncated variants with deletion of any domain of sfcherry-PKM2 were purified and subjected to protein quantification by Coomassie brilliant blue staining. **f** HeLa cells were transfected with full length or truncated variants with deletion of any domain of sfcherry-PKM2 and subjected to relative quantification of SASP mRNA level by Real-time PCR. n = 3, one-way ANOVA was used. *P* = 0.0149 (*CXCL1*: FL vs. Del N), *P* =0.0023 (*CXCL1*: FL vs. Del A1), *P* =0.4960 (*CXCL1*: FL vs. Del B), *P* =0.0043 (*CXCL1*: FL vs. Del C); *P* = 0.0003 (*IL1B*: FL vs Del N), *P* =0.1406 (*IL1B*: FL vs. Del A1), *P* =0.2803 (*IL1B*: FL vs. Del B), *P* =0.0002 (*IL1B*: FL vs. Del C); *P* = 0.2625 (*IL8*: FL vs Del N), *P* =0.0220 (*IL8*: FL vs. Del B), *P* =0.1927 (*IL8*: FL vs. Del C). **g** HEK 293T cells were transfected with full length or truncated variants with deletion of any domain of sfcherry-PKM2 and subjected to detection of p21 protein level by Western blot. **h, i** HeLa cells were transfected with full length or truncated variants with deletion of any domain of sfcherry-PKM2 and subjected to SA-β-gal staining. Representative images were shown in (**h**). Scale bar, 100 μm. The percentage of SA-β-gal positive cells was graphed in (**i**). n = 6, one-way ANOVA was used. *P* = 0.9998 (FL vs. Del N), *P* =0.9972 (FL vs. Del B). **j**, **k** Wild-type Pkm2 and aggregate-prone Pkm2 mutant (A2 domain deletion) were overexpressed in mice via AAV9 system. The body weight of mice was measured every week. **k** After 14 weeks, the mice (**j**) were sacrificed for detection of *p21* and *p16* (left) or *Pkm2* mRNA (left) level in liver by Real-time PCR. n = 5, one-way ANOVA was used. *P* = 0.4940 (Scramble vs. Pkm2 FL), *P* =0.0091 (Scramble vs. Pkm2 Del A2); *P* = 0.8536 (Scramble vs. Pkm2 FL), *P* =0.0366 (Scramble vs. Pkm2 Del A2). NS, not significant, **P* < 0.05, ***P* < 0.01, ****P* < 0.001, *****P* < 0.0001. Error bars represent SEM. All the above experiments were repeated thrice on separate days with similar results. Source data are provided as a Source Data file.

Supplementary Fig. S4 related to Fig. 3

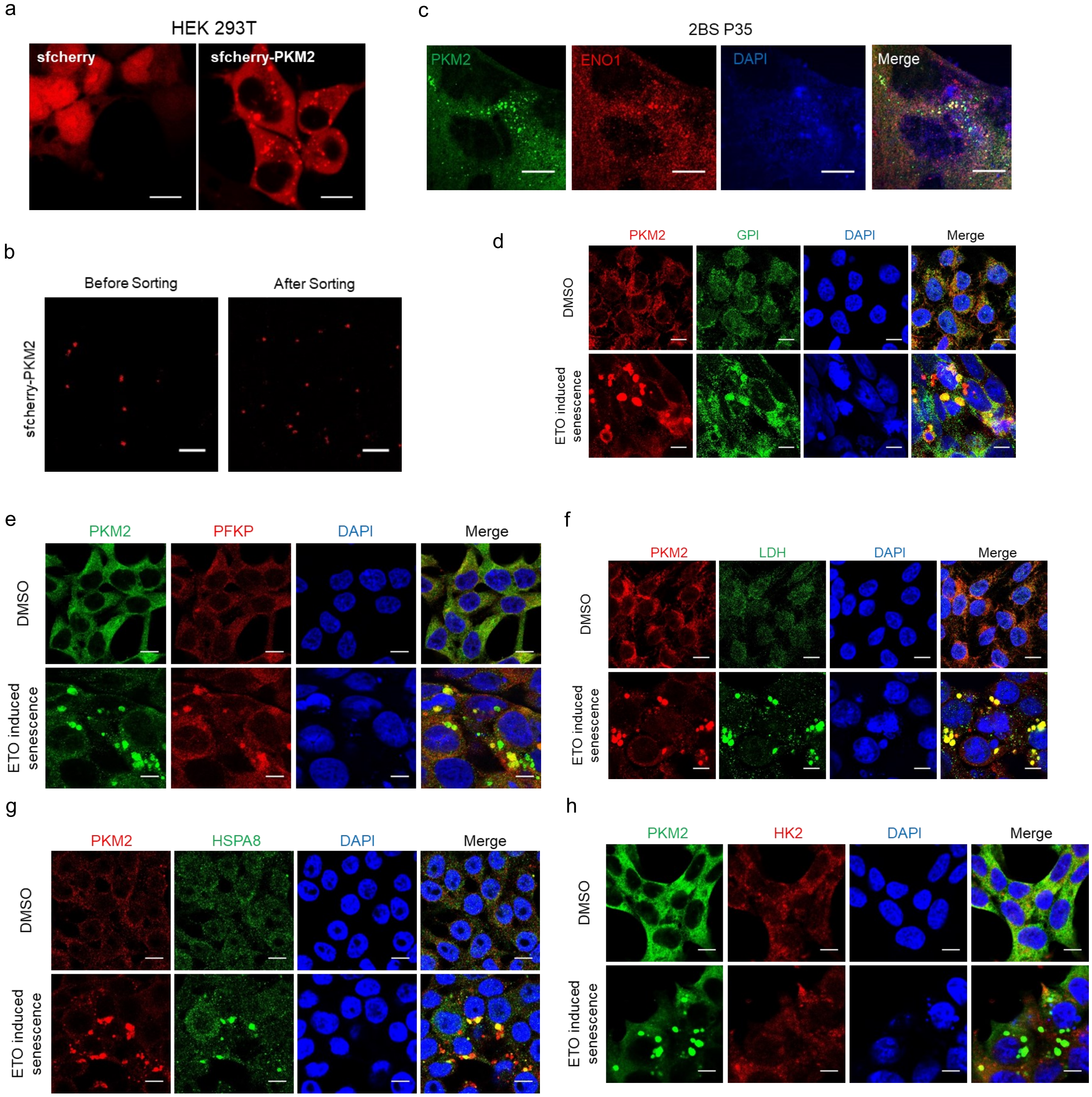

Supplementary Fig. 4 PKM2 aggregates are mainly composed of glycolytic enzymes

a HEK 293T cells were stably expressed with sfcherry or sfcherry-PKM2 and imaged by confocal microscopy. Scale bar, 10 μm. **b** HeLa cells stably expressed sfcherry-PKM2 were collected and lysed followed immunofluorescent imaging of sfhcery-PKM2 before and after FACS. Scale bar, 5 μm. **c** Immunofluorescent staining with PKM2 and ENO1 antibodies was performed in old fibroblasts 2BS (P35). Scale bar, 20 μm. **d** Senescent HEK 293T cells were induced by 2 μM etoposide (ETO) and subjected to immunofluorescent imaging of PKM2 and GPI. Scale bar, 10 μm. **e** Senescent HEK 293T cells were induced by 2 μM etoposide (ETO) and subjected to immunofluorescent imaging of PKM2 and PFKP. Scale bar, 10 μm. **f** Senescent HEK 293T cells were induced by 2 μM etoposide (ETO) and subjected to immunofluorescent imaging of PKM2 and LDH. Scale bar, 10 μm. **g** Senescent HEK 293T cells were induced by 2 μM etoposide (ETO) and subjected to immunofluorescent imaging of PKM2 and HSPA8. Scale bar, 10 μm. **h** Senescent HEK 293T cells were induced by 2 μM etoposide (ETO) and subjected to immunofluorescent imaging of PKM2 and HK2. Scale bar, 10 μm. All the above experiments were repeated thrice on separate days with similar results.

Supplementary Fig. S5 related to Fig. 4

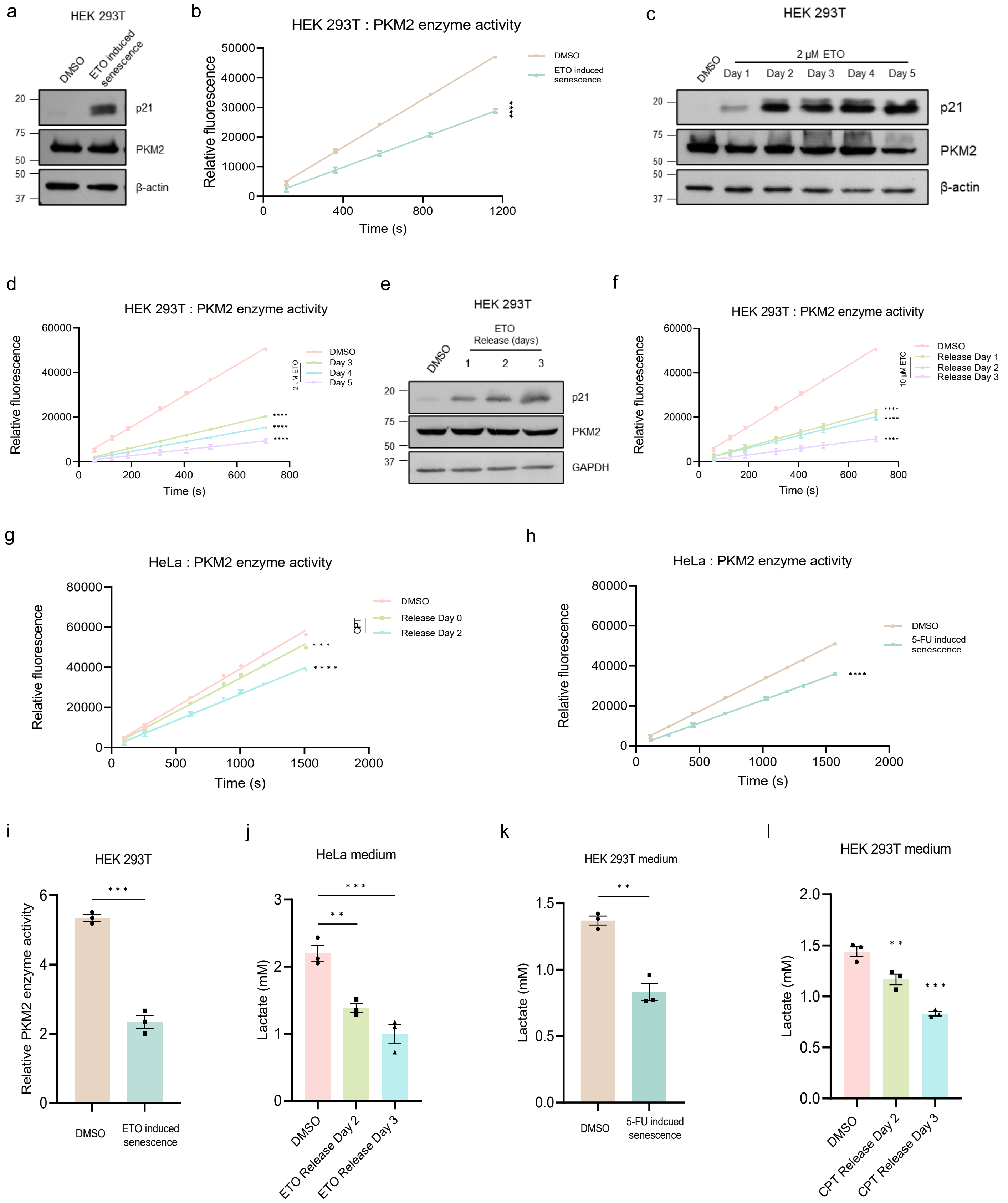

Supplementary Fig. 5 PKM2 enzymatic activity and glycolytic flux are dampened in senescent cells

a, **b** HEK 293T cells were treated with 2 μM etoposide for three days to induce senescence followed by relative quantification of p21, PKM2 protein level by Western blot (**a**) and measurement of PKM2 enzymatic activity via LDH-coupled kinetic assay (**b**). n = 3, Two-tailed unpaired t-test was used. **c**, **d** HEK 293T cells were treated with 2 μM etoposide for indicated days followed by Western blot of p21, PKM2 (**c**) and measurement of PKM2 enzymatic activity via LDH-coupled kinetic assay (**d**). n = 3, one-way ANOVA was used. **e**, **f** HEK 293T cells were treated with 10 μM etoposide and released for indicated days followed by Western blot of p21, PKM2 (**e**) and measurement of PKM2 enzymatic activity via LDH-coupled kinetic assay (**f**). n = 3, one-way ANOVA was used. **g** HeLa cells were treated with 50 nM CPT and released for indicated days followed by measurement of PKM2 enzymatic activity via LDH-coupled kinetic assay. n = 3, one-way ANOVA was used. P = 0.0001 (DMSO vs. Release Day 0). **h** HeLa cells were treated with 100 μM 5-FU and released for indicated days followed by measurement of PKM2 enzymatic activity via LDH-coupled kinetic assay. n = 3, Two-tailed unpaired t-test was used. **i** HEK 293T cells were treated with 10 μM etoposide for three days followed by measurement of PKM2 enzymatic activity with a commercial kit (K709-100). n = 3, Two-tailed unpaired t-test was used. P = 0.0001. **j** HeLa cells were treated with 10 μM etoposide and released for indicated days followed by quantification of lactate in the culture medium. n = 3, one-way ANOVA was used. P = 0.0039 (DMSO vs. ETO Release Day 2), P = 0.0005 (DMSO vs. ETO Release Day 3). **k** HEK 293T cells were treated with 100 μM 5-FU and released for three days followed by quantification of lactate in the culture medium. n = 3, Two-tailed unpaired t-test was used. P = 0.0018. **l** HEK 293T cells were treated with 25 nM CPT and released for indicated days followed by quantification of lactate in the culture medium. n = 3, one-way ANOVA was used. P = 0.0080 (DMSO vs. CPT Release Day 2), P = 0.0001 (DMSO vs. CPT Release Day 3). $^{**}P < 0.01$, $^{***}P < 0.001$, $^{****}P < 0.0001$. Error bars represent SEM. Source data are provided as a Source Data file.

Supplementary Fig. S6 related to Fig. 5

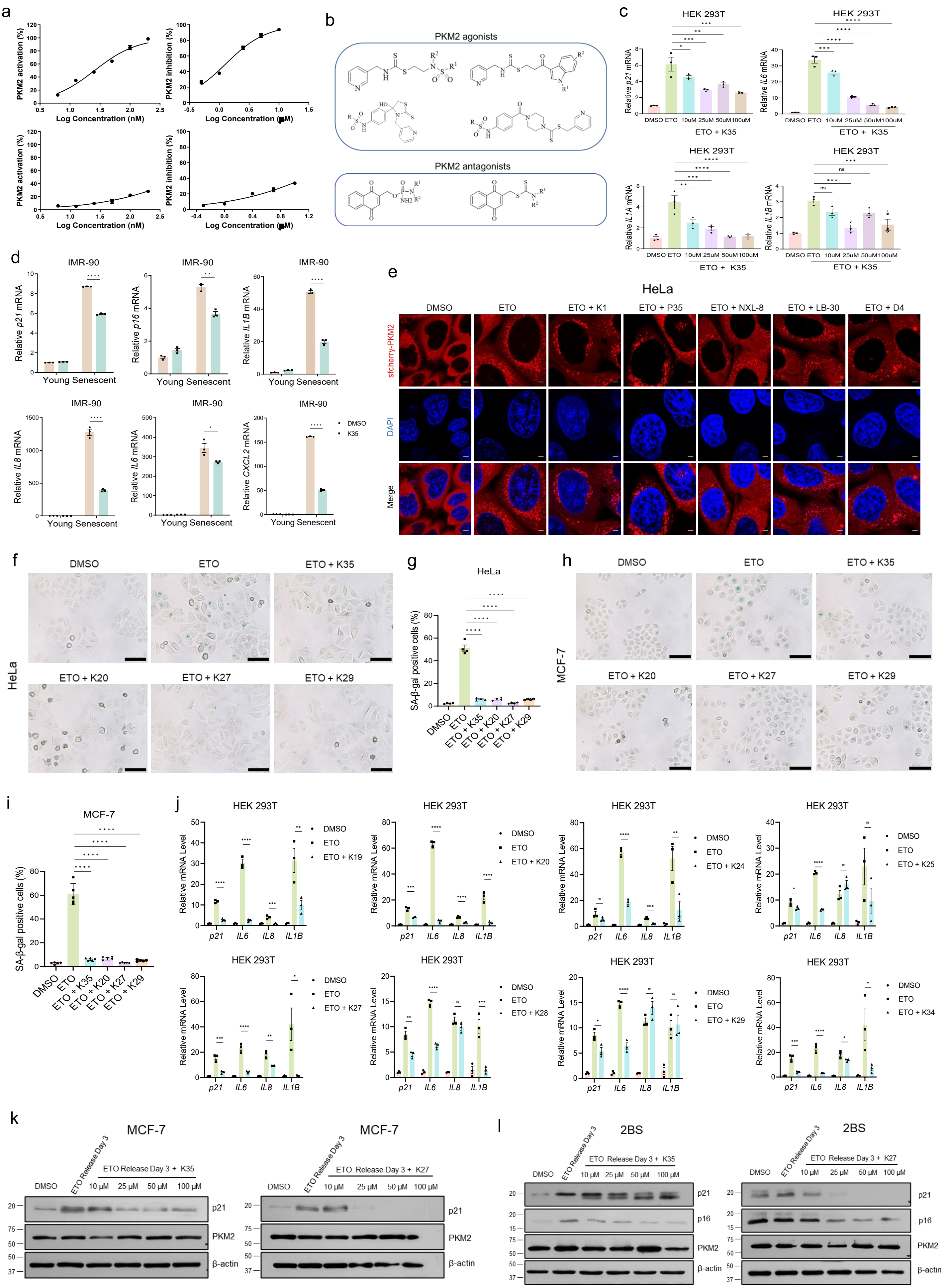

Supplementary Fig. 6 K35 and its analogs alleviate cellular senescence

a The effects of in-house molecule library to PKM2 enzymatic activity determined by LDH-coupled kinetic assay. **b** The chemical structure of PKM2 agonists and antagonists for screening for dissolution agents of PKM2 aggregates. **c** qPCR analysis of *p21*, SASP mRNA level in HEK 293T treated with 10 μM etoposide for 24 hours and released for three days in combination with concentration gradient of K35. n = 3, one-way ANOVA was used. *P* = 0.0409 (*p21*: ETO vs. 10 μM), *P* = 0.0003 (*p21*: ETO vs. 25 μM), *P* = 0.0022 (*p21*: ETO vs. 50 μM), *P* = 0.0001 (*p21*: ETO vs. 50 μM); *P* = 0.0004 (*IL6*: ETO vs. 10 μM); *P* = 0.0034 (*IL1A*: ETO vs. 10 μM), *P* = 0.0005 (*IL1A*: ETO vs. 25 μM); *P* = 0.0983 (*IL1B*: ETO vs. 10 μM), *P* = 0.0003 (*IL1B*: ETO vs. 25 μM), *P* = 0.0781 (*IL1B*: ETO vs. 50 μM), *P* = 0.0010 (*IL1B*: ETO vs. 50 μM). **d** qPCR analysis of *p21*, *p16* and SASP mRNA level in young or senescent fibroblasts IMR-90 exposed to DMSO or K35 (25 μM). n = 3, Two-tailed unpaired t-test was used. *P* = 0.0010 (*p16*), *P* = 0.0386 (*IL6*). **e** Immunofluorescent imaging of PKM2 aggregates in HeLa stably expressed sfcherry-PKM2 were treated with 2 μM etoposide together with DMSO or indicated compounds (50 μM) for two days. Scale bar, 5 μm. This experiment were repeated thrice with similar results. **f**, **g** Representative images (**f**) and quantification (**g**) of SA-β-gal staining in HeLa treated with 10 μM etoposide for 24 hours and released for three days together with DMSO, K35 or K35 analogs. Scale bar, 100 μm. n = 4, one-way ANOVA was used. **h**, **i** Representative images (**h**) and quantification (**i**) of SA-β-gal staining in MCF-7 treated with 20 μM etoposide for 24 hours and released for three days together with DMSO, K35 or K35 analogs. Scale bar, 100 μm. n = 5, one-way ANOVA was used. **j** qPCR analysis of *p21*, SASP mRNA level in HEK 293T treated with etoposide (10 μM) for 24 hours and released for three days together with DMSO or K35 analogs (100 μM). n = 3, one-way ANOVA was used. *P* = 0.0093 (*IL1B*: ETO vs. ETO + K19), *P* = 0.0007 (*IL8*: ETO vs. ETO + K19); *P* = 0.0003 (*p21*: ETO vs. ETO + K20); *P* = 0.0842 (*p21*: ETO vs. ETO + K24), *P* = 0.0002 (*IL8*: ETO vs. ETO + K24), *P* = 0.0091 (*IL1B*: ETO vs. ETO + K24)*;* *P* = 0.0499 (*p21*: ETO vs. ETO + K25), *P* = 0.1317 (*IL8*: ETO vs. ETO + K25), *P* = 0.1728 (*IL1B*: ETO vs. ETO + K25);*P* = 0.0003 (*p21*: ETO vs. ETO + K27), *P* = 0.0012 (*IL8*: ETO vs. ETO + K27), *P* = 0.0143 (*IL1B*: ETO vs. ETO + K27); *P* = 0.0014 (*p21*: ETO vs. ETO + K28), *P* = 0.1584 (*IL8*: ETO vs. ETO + K28), *P* = 0.0010 (*IL1B*: ETO vs. ETO + K28); *P* = 0.0137 (*p21*: ETO vs. ETO + K29), *P* = 0.1048 (*IL8*: ETO vs. ETO + K29), *P* = 0.9451 (*IL1B*: ETO vs. ETO + K29); *P* = 0.0003 (*p21*: ETO vs. ETO + K34), *P* = 0.0306 (*IL8*: ETO vs. ETO + K34), *P* = 0.0288 (*IL1B*: ETO vs. ETO + K34). **k** Immunoblotting of p21, PKM2 level in MCF-7 treated with 20 μM etoposide for 24 hours and released for three days together with concentration gradient of K35 (left) or K27 (right). **l** Immunoblotting of p21, PKM2 level in young fibroblasts 2BS treated with 20 μM etoposide for 24 hours and released for three days together with concentration gradient of K35 (left) or K27 (right). *$P < 0.05$, **$P < 0.01$, ***$P < 0.001$, ****$P < 0.0001$. Error bars represent SEM.

Supplementary Fig. S7 related to Fig. 5

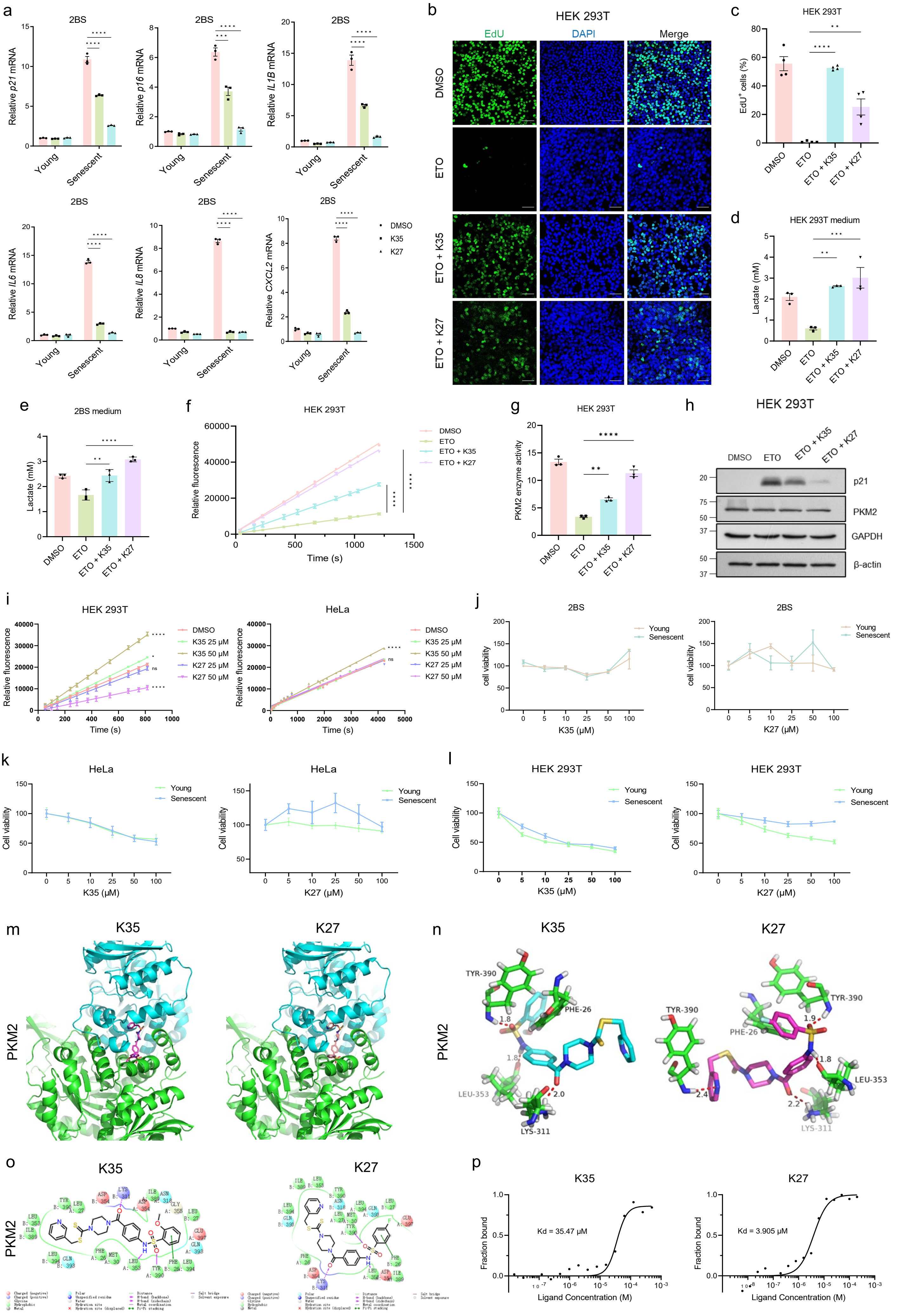

Supplementary Fig. 7 K35 and K27 rescue PKM2 enzymatic activity and glycolytic flux

a qPCR analysis of *p21*, *p16* and SASP level in young or senescent 2BS exposed to DMSO, 25 μM K35/K27. n = 3. *P* = 0.0004 (*p16*: DMSO vs. K35). **b**, **c** Representative images (**b**) and quantification (**c**) of EdU staining proliferation assay in HEK 293T treated with 10 μM etoposide for 24 hours and released for three days together with DMSO, 50 μM K35/K27. Scale bar, 50 μm. n = 4. *P* = 0.0017 (ETO vs. ETO + K27). **d**, **e** Quantification of lactate in culture medium of HEK 293T exposed to 10 μM etoposide (**d**) or 2BS exposed to 20 μM etoposide (**e**) for 24 hours and released for three days together with DMSO, 50 μM K35/K27. n = 3. *P* = 0.0015 (HEK293T: ETO vs. ETO + K35), *P* = 0.0005 (HEK293T: ETO vs. ETO + K27). *P* = 0.0013 (2BS: ETO vs. ETO + K35). **f** Measurement of PKM2 enzymatic activity via LDH-coupled kinetic assay in HEK 293T exposed to 10 μM etoposide for 24 hours and released for three days together with DMSO, 50 μM K35/K27. n = 3. **g** Measurement of PKM2 enzymatic activity with commercial kit (K709-100) in HEK 293T exposed to 10 μM etoposide for 24 hours and released for three days together with DMSO, 50 μM K35/K27. n = 3. *P* = 0.0017 (ETO vs. ETO + K35). **h** Immunoblotting of p21, PKM2 in HEK 293T exposed to 10 μM etoposide for 24 hours and released for three days together with DMSO, 50 μM K35/K27. **i** Measurement of PKM2 enzymatic activity via LDH-coupled kinetic assay in HEK 293T (left) or HeLa (right) treated with DMSO, 50 μM K35/K27 for two days. n = 3. *P* = 0.0411 (HEK 293T: DMSO vs. K35 25 μM), *P* = 0.3465 (HEK 293T: DMSO vs. K27 25 μM); *P* = 0.8277 (HeLa: DMSO vs. K35 25 μM), *P* = 0.1844 (HeLa: DMSO vs. K27 25 μM), *P* = 0.9746 (HeLa: DMSO vs. K27 50 μM). **j** CCK-8 analysis of 2BS exposed to K35 (left) or K27 (right) for three days. n = 3. **k**, **l** CCK-8 analysis of HeLa (**k**) or HEK 293T (**l**) exposed to 10 μM etoposide for 24 hours and released for two days together with 50 μM K35 (left) or K27 (right) for another two days, n = 5. **m**-**o** Molecular docking of PKM2 (PDB: 4G1N) with K35 (left) or K27 (right). **p** MST assay of PKM2 with K35 (left) or K27 (right). **P* < 0.05, ***P* < 0.01, ****P* < 0.001, *****P* < 0.0001. one-way ANOVA. Error bars represent SEM. These experiments were repeated thrice with similar results. Source data are provided as a Source Data file.

Supplementary Fig. S8 related to Fig. 6

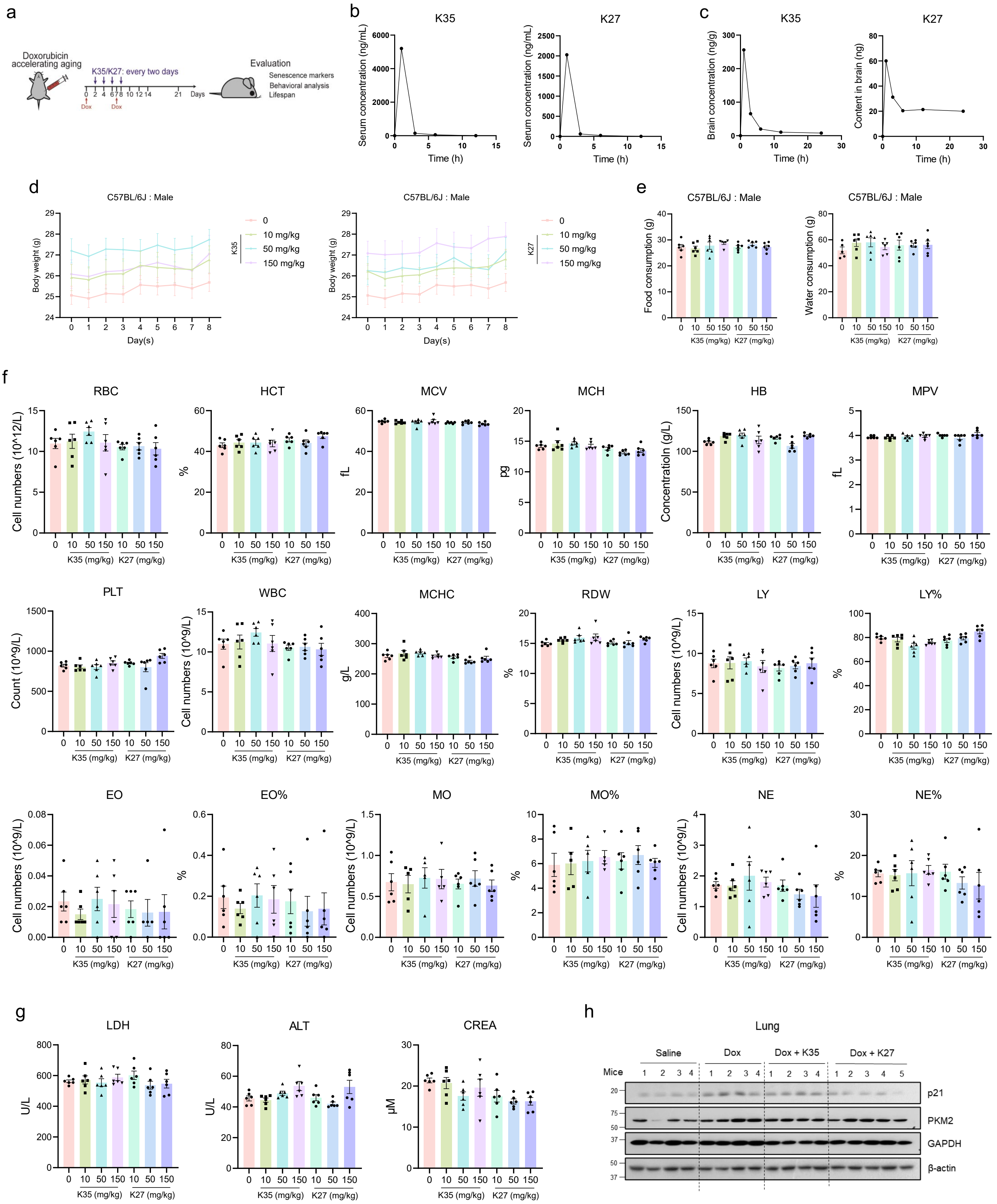

Supplementary Fig. 8 K35 and K27 ameliorate aging phenotypes

a The administration model of doxorubicin-induced premature mice. C57BL/6J mice (male, 8 weeks) were injected intraperitoneally with saline or doxorubicin (10 mg/kg) twice at day 0 and day 7. From day 2, the mice exposed to doxorubicin were intragastrically administrated with vehicle or K35 (50 mg/kg) or K27 (50 mg/kg) every two days. **b**, **c** Male C57BL/6J (8 weeks) were intragastrically administrated with 50 mg/kg K35 or K27. After indicated hours, mice were sacrificed to quantify K35 or K27 content in serum or brain by LC-MS. **d**, **e** Male C57BL/6J (8 weeks) were intragastrically administrated with different dosage of K35 or K27 consecutively for 8 days. During the period, the body weight (**d**), food and water consumption (**e**) were recorded. n = 6. **f**, **g** At the last day, the mice (**d**) were sacrificed for routine blood tests (**f**) and examinations of LDH, ALT, CREA in serum (**g**). n = 6. **h** Mice underwent the procedure described in (**a**). At day 20, the mice were sacrificed for lung tissue which were grinded in liquid nitrogen for detection of p21, PKM2 protein level by Western blot. All the above experiments were repeated thrice on separate days with similar results. Source data are provided as a Source Data file.

Supplementary Fig. S9 related to Fig. 6

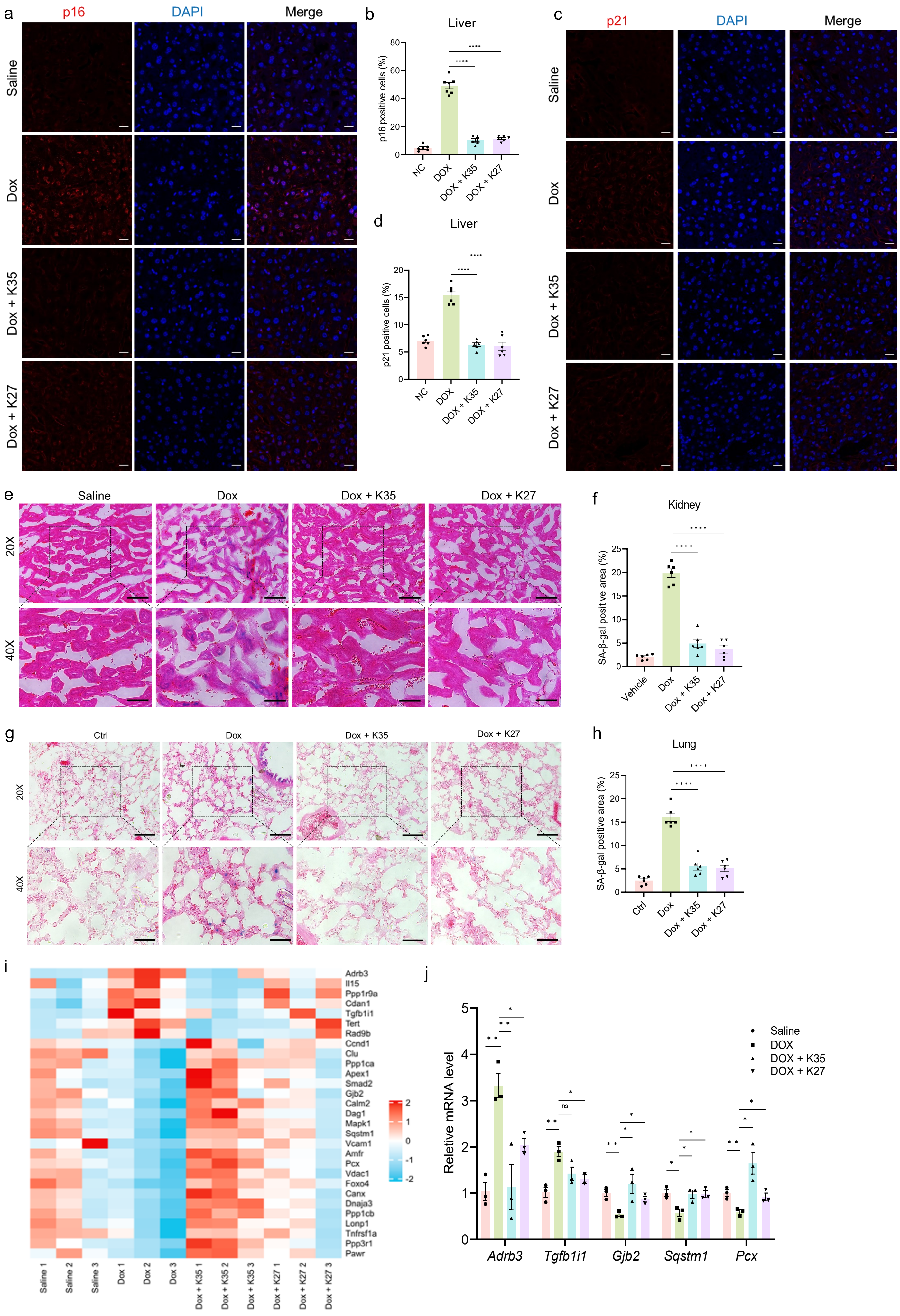

Supplementary Fig. 9 K35 and K27 ameliorate aging phenotypes

a-**d** C57BL/6J mice (male, 8 weeks) were injected intraperitoneally with saline or doxorubicin (10 mg/kg) twice at day 0 and day 7. From day 2, the mice exposed to doxorubicin were intragastrically administrated with vehicle or K35 (50 mg/kg) or K27 (50 mg/kg) every two days. At day 20, the mice were sacrificed for liver tissue which were embedded in OCT for frozen tissue sections followed by immunofluorescent imaging of p16 (**a**) or p21 (**c**). Scale bar, 20 μm. The percentage of p16 (**b**) or p21 (**d**) positive cells were counted separately. n = 6-7, one-way ANOVA was used. **e**-**h** Mice underwent the same procedure as in (**a**). At day 20, the mice were sacrificed for kidney (**e**) and lung (**g**) tissue which were embedded in OCT for frozen tissue sections followed by SA-β-gal staining. Scale bar, 100 μm for 20X magnification images. The percentage of SA-β-gal positive areas in kidney (**f**) or lung (**h**) were counted separately. n = 6, one-way ANOVA was used. **i**, **j** Mice underwent the same procedure as in (**a**). At day 20, the mice were sacrificed for liver tissue which were subjected to RNA sequencing. The senescence or aging-related genes (KEGG or GO annotations) were shown in (**i**). The fold changes of senescence or aging-related genes were verified by Real-time PCR (**j**). n = 3, one-way ANOVA was used. P = 0.0016 (*Adrb3*: Saline vs. DOX), P = 0.0022 (*Adrb3*: DOX vs. DOX + K35), P = 0.0401 (*Adrb3*: DOX vs. DOX + K27); P = 0.0028 (*Tgfb1i1*: Saline vs. DOX), P = 0.0579 (*Tgfb1i1*: DOX vs. DOX + K35), P = 0.0385 (*Tgfb1i1*: DOX vs. DOX + K27); P = 0.0057 (*Gjb2*: Saline vs. DOX), P = 0.0350 (*Gjb2*: DOX vs. DOX + K35), P = 0.0109 (*Gjb2*: DOX vs. DOX + K27); P = 0.0112 (*Sqstm1*: Saline vs. DOX), P = 0.0151 (*Sqstm1*: DOX vs. DOX + K35), P = 0.0146 (*Sqstm1*: DOX vs. DOX + K27); P = 0.0087 (*Pcx*: Saline vs. DOX), P = 0.0114 (*Pcx*: DOX vs. DOX + K35), P = 0.0224 (*Pcx*: DOX vs. DOX + K27). *$P < 0.05$, **$P < 0.01$, ****$P < 0.0001$. Error bars represent SEM.

Supplementary Fig. S10 related to Fig. 6

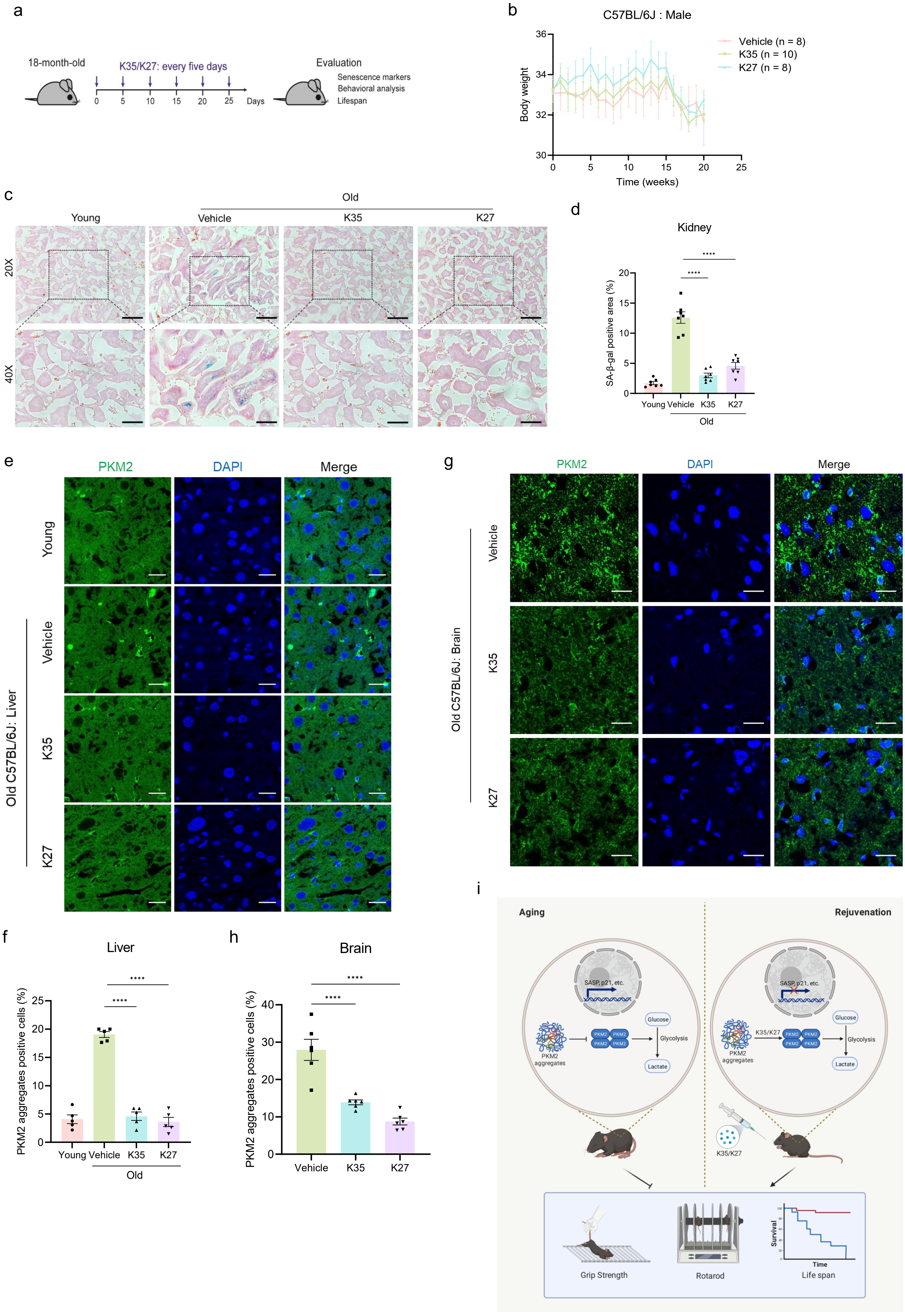

Supplementary Fig. 10 K35 and K27 ameliorate aging phenotypes

a The administration model of 18-month-old mice with vehicle or K35 or K27. 18-month-old C57BL/6J were administrated intragastrically with vehicle or K35 (50 mg/kg) or K27 (50 mg/kg) every five days. **b** Body weight of the mice described in (**a**) were recorded once a week, n = 8-10. **c**, **d** Mice were treated as in (**a**). After six months, kidney of young and old mice was prepared as frozen sections for SA-β-gal staining. Images were shown in (**c**). Scale bar, 100 μm for 20X magnification images. The percentage of SA-β-gal positive areas were counted in (**d**). n = 7, one-way ANOVA was used. **e**-**h** Mice underwent the same procedure as in (**a**). After six months, the mice were sacrificed for liver (**e**) or brain (**g**) which was prepared as frozen sections for fluorescent imaging of PKM2. Scale bar, 100 μm. The percentage of cells with PKM2 aggregates in liver (**f**) or brain (**h**) were counted separately. n = 5-6, one-way ANOVA was used. **i** The graphic model of the manuscript (Created with BioRender.com, released under a Creative Commons Attribution-NonCommercial-NoDerivs 4.0 International license). ****$P < 0.0001$. Error bars represent SEM.

Table S1 The primers used for qPCR

Gene Name	Forward primers	Reverse primers
Human *p21*	AGGTGGACCTGGAGACTCTCAG	TCCTCTTGGAGAAGATCAGCCG
Human *p16*	CTCGTGCTGATGCTACTGAGGA	GGTCGGCGCAGTTGGGCTCC
Human *PKM2*	ATGGCTGACACATTCCTGGAGC	CCTTCAACGTCTCCACTGATCG
Human *IL1A*	TGTATGTGACTGCCCAAGATGAAG	AGAGGAGGTTGGTCTCACTACC
Human *IL1B*	CCACAGACCTTCCAGGAGAATG	GTGCAGTTCAGTGATCGTACAGG
Human *IL8*	AAGGAAAACTGGGTGCAGAG	ATTGCATCTGGCAACCCTAC
Human *IL6*	AGACAGCCACTCACCTCTTCAG	TTCTGCCAGTGCCTCTTTGCTG
Human *CXCL2*	GGCAGAAAGCTTGTCTCAACCC	CTCCTTCAGGAACAGCCACCAA
Human *GAPDH*	GAGTCAACGGATTTGGTCGT	TTGATTTTGGAGGGATCTCG
Human *β-actin*	GGACTTCGAGCAAGAGATGG	AGGAAGGAAGGCTGGAAGAG
Mouse *Cxcl2*	CATCCAGAGCTTGAGTGTGACG	GGCTTCAGGGTCAAGGCAAACT
Mouse *p21*	AGCAAAGTGTGCCGTTGTCT	AGAAATCTGTCAGGCTGGTC
Mouse *p16*	CTCAGCCCGCCTTTTTCTTC	CGCCTTCGCTCAGTTTCTCATG
Mouse *Il1α*	ACGGCTGAGTTTCAGTGAGACC	CACTCTGGTAGGTGTAAGGTGC
Mouse *Il1β*	TGGACCTTCCAGGATGAGGACA	GTTCATCTCGGAGCCTGTAGTG
Mouse *Il6*	TACCACTTCACAAGTCGGAGGC	CTGCAAGTGCATCATCGTTGTTC
Mouse *TNFα*	GGTGCCTATGTCTCAGCCTCTT	GCCATAGAACTGATGAGAGGGAG
Mouse *Adrb3*	AGGCACAGGAATGCCACTCCAA	GCTTAGCCACAACGAACACTCG
Mouse *Tgfb1i1*	GGTCTGGAGAATCTTCAGGAACC	CACCACTGGAAGAGGAGAATGG
Mouse *Gjb2*	ATCTGGCTCACGGTCCTCTTCA	GGAAGTGGTGGTCGTAGCATAC
Mouse *Sqstm1*	GCTCTTCGGAAGTCAGCAAACC	GCAGTTTCCCGACTCCATCTGT
Mouse *Pcx*	GGATGACCTCACAGCCAAGCAT	GCAATCGAAGGCTGCGTACAGT
Mouse *Gapdh*	GTGGCAAAGTGGAGATTGTTG	AGTCTTCTGGGTGGCAGTGAT
Mouse *β-actin*	CATTGCTGACAGGATGCAGAAGG	TGCTGGAAGGTGGACAGTGAGG

Table S2 The materials used in the paper

REAGENT or RESOURCE	SOURCE	IDENTIFIER
Antibodies		
Mouse anti-PFKP	Santa Cruz Biotechnology	Cat#sc-514824
Rabbit anti-PKM2	Cell Signaling Technology	Cat#4053; RRID: AB_1904096
Mouse anti-PKM2	Santa Cruz Biotechnology	Cat#sc-365684; RRID: AB_10844484
Mouse anti-ENO1	Santa Cruz Biotechnology	Cat#sc-271384; RRID: AB_10609119
Rabbit anti-PGK1	Abcam	Cat#ab199438
Rabbit anti-GPI	Abcam	Cat#ab167394; RRID: AB_2736921
Mouse anti-p53	Santa Cruz Biotechnology	Cat#sc-126; RRID: AB_628082
Mouse anti-p21	Santa Cruz Biotechnology	Cat#sc-6246; RRID: AB_628073
Rabbit anti-p21	Cell Signaling Technology	Cat#2947; RRID: AB_823586
Rabbit anti-p16	Zen-BIO	Cat#380963
Mouse anti-p16	Santa Cruz Biotechnology	Cat#sc-1661; RRID: AB_628067
Rabbit anti-LC3	Sigma-Aldrich	Cat#L8918; RRID: AB_1079382
Rabbit anti-LAMP2a	Zen bio	Cat#R24835; RRID:
Rabbit anti-CLAR	Zen bio	Cat#380959; RRID:
Rabbit anti-GAPDH	Cell Signaling Technology	Cat#5174; RRID: AB_10622025
Rabbit anti-PEX19	Zen bio	Cat#389339
Rabbit anti-p70S6K	Cell Signaling Technology	Cat#9202; RRID: AB_331676
Mouse anti-β-actin	Santa Cruz Biotechnology	Cat#sc-8432; RRID: AB_626630
Mouse anti-PCNA	Zen bio	Cat#200947-2E1; RRID: AB_2722718
Rabbit anti-VDAC	Cell Signaling Technology	Cat#4866; RRID: AB_2272627
Rabbit anti-HSPA8	HUABIO	Cat#ET1602-33
Mouse anti–HK2	Abcam	Cat#ab104836; AB_10710018
Mouse anti–HA	Thermo Fisher Scientific	Cat#26183; RRID: AB_2533052
Donkey anti-rabbit Alexa Fluor 488	Thermo Fisher Scientific	Cat#A-21206; RRID: AB_2535792
Donkey anti-mouse Alexa Fluor 594	Thermo Fisher Scientific	Cat#A-21203; RRID: AB_141633
Anti–PHGDH	Santa Cruz Biotechnology	Cat#sc-100317; RRID: AB_2165393
Anti–MTHFD1	Santa Cruz Biotechnology	Cat#sc-271412; RRID: AB_10611082
Anti-LAMP-2	BioLegend	Cat#354301; RRID: AB_11204081

Bacterial and virus strains		
TSurbo Chemically Competent Cell	Tsingke Biotechnology	Cat#TSC-C02
TSsetta Chemically Competent Cell	Tsingke Biotechnology	Cat#TSC-E04
Chemicals, peptides, and recombinant proteins		
Puromycin	Sigma-Aldrich	Cat#P8833
DAPI	Sigma-Aldrich	Cat#D9542
Hoechst 33342	Solarbio	Cat#C0031
Camptothecin	Selleck	Cat# S1288
5-Fluorouracil	Sigma-Aldrich	Cat#F6627
Etoposide	Sigma-Aldrich	Cat#E1383
$AgNO3$	Thermo Fisher Scientific	Cat#S486
TRI Reagent	Sigma-Aldrich	Cat#T9424
Pierce™ Anti-HA Magnetic Beads	Thermo Fisher Scientific	Cat#88836
Antigen Retrieval Solution	Solarbio	Cat#C1035
H_2O_2	Sigma-Aldrich	Cat#323381
Critical commercial assays		
Senescence β-Galactosidase Staining Kit	Cell Signaling Technology	Cat#9860
BeyoClick™ EdU Cell Proliferation Kit with Alexa Fluor 488	Beyotime	Cat#C0071S
Pyruvate Kinase Activity Colorimetric/Fluorometric Assay Kit	BioVision	Cat#K709-100
CheKine™ Micro Lactate Assay Kit	Abbkine	Cat#KTB1100
Cell Counting Kit-8	Dojindo	Cat#CK04
First Strand cDNA Synthesis kit	Yeasen	Cat#11123ES10
Hieff® qPCR SYBR Green Master Mix	Yeasen	Cat#11202ES03
Experimental models: Cell lines		
Human: HEK293T	ATCC	Cat#CRL-11268
Human: 2BS	Zebin Mao (Peking University)	N/A
Human: HeLa	Qing Chang (Peking University)	N/A
Human: MCF-7	ATCC	Cat#HTB-22
Experimental models: Organisms/strains		
Mouse: C57BL/6J		
Oligonucleotides		
Human PKM2 shRNA #1: 5'-CCGGGGGTGAACTTTGC CATGAATGCTCGAGCATTCATGGCAAAGTTCACCCT TTTTG - 3'	Tsingke Biotechnology	N/A
Human PKM2 shRNA #2: 5'- CCGGTCATTGCTGTGACCCGGAATCCTCGAGGATT CCGGGTCACAGCAATGATTTTT G - 3'	Tsingke Biotechnology	N/A
Recombinant DNA		
pHBLV-TMEM192-3HA	This manuscript	N/A
pHBLV-TMEM192-2Flag	This manuscript	N/A
pHBLV-sfcherry	This manuscript	N/A

pLVX-EnCMV-HRAS(human)-G12V-PGK-Puro	MIAOLING BIOLOGY	Cat#P34482
pHBLV-sfcherry-PKM2	This manuscript	N/A
pHBLV-sfcherry-PKM2 Del N (residue 1-44)	This manuscript	N/A
pHBLV-sfcherry-PKM2 Del A1 (residue 45-166)	This manuscript	N/A
pHBLV-sfcherry-PKM2 Del B1 (residue 167-218)	This manuscript	N/A
pHBLV-sfcherry-PKM2 Del A2 (residue 219-389)	This manuscript	N/A
pHBLV-sfcherry-PKM2 Del C (residue 390-531)	This manuscript	N/A
pLKO.1-puro-shRNA-PKM2 #1	This manuscript	N/A
pLKO.1-puro-shRNA-PKM2 #2	This manuscript	N/A
Software and algorithms		
Prism 9	GraphPad	https://www.graphpad.com/scientificsoftware/prism/
ZEN 3.2	Zeiss	https://www.zeiss.com
ImageJ	NIH	https://imagej.nih.gov/ij/
FlowJo v10.6.2	BD	https://www.flowjo.com/solutions/flowjo/downloads
Adobe Illustrator	Adobe	https://www.adobe.com/products/illustrator/free-trial-download.html
