# Supplementary material for: PKM2 aggregation drives metabolism reprograming during aging process
Source: Nat Commun. 2024 Jul 9;15:5761. doi: 10.1038/s41467-024-50242-y (PMC11233639; doi:10.1038/s41467-024-50242-y)
Supplement: Supplementary file 3 — Reporting Summary [file 41467_2024_50242_MOESM3_ESM.pdf]

nature portfolio

Corresponding author(s): Juanyuan Luo
Last updated by author(s): Jun 24, 2024

Reporting Summary

Nature Portfolio wishes to improve the reproducibility of the work that we publish. This form provides structure for consistency and transparency in reporting. For further information on Nature Portfolio policies, see our Editorial Policies and the Editorial Policy Checklist.

Statistics

For all statistical analyses, confirm that the following items are present in the figure legend, table legend, main text, or Methods section.

n/a	Confirmed	
[ ]	[x]	The exact sample size (*n*) for each experimental group/condition, given as a discrete number and unit of measurement
[ ]	[x]	A statement on whether measurements were taken from distinct samples or whether the same sample was measured repeatedly
[ ]	[x]	The statistical test(s) used AND whether they are one- or two-sided<br>*Only common tests should be described solely by name; describe more complex techniques in the Methods section.*
[x]	[ ]	A description of all covariates tested
[x]	[ ]	A description of any assumptions or corrections, such as tests of normality and adjustment for multiple comparisons
[ ]	[x]	A full description of the statistical parameters including central tendency (e.g. means) or other basic estimates (e.g. regression coefficient) AND variation (e.g. standard deviation) or associated estimates of uncertainty (e.g. confidence intervals)
[ ]	[x]	For null hypothesis testing, the test statistic (e.g. *F*, *t*, *r*) with confidence intervals, effect sizes, degrees of freedom and *P* value noted<br>*Give P values as exact values whenever suitable.*
[x]	[ ]	For Bayesian analysis, information on the choice of priors and Markov chain Monte Carlo settings
[x]	[ ]	For hierarchical and complex designs, identification of the appropriate level for tests and full reporting of outcomes
[x]	[ ]	Estimates of effect sizes (e.g. Cohen's *d*, Pearson's *r*), indicating how they were calculated

Our web collection on statistics for biologists contains articles on many of the points above.

Software and code

Policy information about availability of computer code

Data collection	Real Time quantitative PCR data750LTQ Orbitrap Velos0 Real-time PCR System (Applied Biosystems); RNA sequencing data: Illumina NovaSeq 6000; Mass spectrum: LTQ Orbitrap Velos; Flow cytometry: BD FACSymphony™ S6.
Data analysis	Graphpad Prism 9; Carl Zeiss ZEN 3.2 (blue edition); MaxQuant; ImageJ; FlowJo v10.6.2.

For manuscripts utilizing custom algorithms or software that are central to the research but not yet described in published literature, software must be made available to editors and reviewers. We strongly encourage code deposition in a community repository (e.g. GitHub). See the Nature Portfolio guidelines for submitting code & software for further information.

Data

Policy information about availability of data

All manuscripts must include a data availability statement. This statement should provide the following information, where applicable:

- Accession codes, unique identifiers, or web links for publicly available datasets
- A description of any restrictions on data availability
- For clinical datasets or third party data, please ensure that the statement adheres to our policy

All related raw sequencing and processed data have been deposited at the Gene Expression Omnibus under accession number GSE268773. All other data are available in the main text or in the supplemental information. The uncropped and unprocessed scans of all the blots are supplied in the Source Data file.

Research involving human participants, their data, or biological material

Policy information about studies with human participants or human data. See also policy information about sex, gender (identity/presentation), and sexual orientation and race, ethnicity and racism.

Reporting on sex and gender	N/A
Reporting on race, ethnicity, or other socially relevant groupings	N/A
Population characteristics	N/A
Recruitment	N/A
Ethics oversight	N/A

Note that full information on the approval of the study protocol must also be provided in the manuscript.

Field-specific reporting

Please select the one below that is the best fit for your research. If you are not sure, read the appropriate sections before making your selection.

- [x] Life sciences
- [ ] Behavioural & social sciences
- [ ] Ecological, evolutionary & environmental sciences

For a reference copy of the document with all sections, see nature.com/documents/nr-reporting-summary-flat.pdf

Life sciences study design

All studies must disclose on these points even when the disclosure is negative.

Sample size	No specific statistical measure was taken to decide sample size. Minimum sample sizes were predetermined from power estimate based on pilot experiments.
Data exclusions	No data has been excluded from analysis.
Replication	All attempts at replication were successful. Figure legends state how many times each experiment was performed.
Randomization	The allocation of mice was randomly.
Blinding	Blinding was not done since the study relies on the investigator's treatment on cells or mice.

Reporting for specific materials, systems and methods

We require information from authors about some types of materials, experimental systems and methods used in many studies. Here, indicate whether each material, system or method listed is relevant to your study. If you are not sure if a list item applies to your research, read the appropriate section before selecting a response.

Materials & experimental systems

n/a	Involved in the study	
[ ]	[x]	Antibodies
[ ]	[x]	Eukaryotic cell lines
[x]	[ ]	Palaeontology and archaeology
[ ]	[x]	Animals and other organisms
[x]	[ ]	Clinical data
[x]	[ ]	Dual use research of concern
[x]	[ ]	Plants

Methods

n/a	Involved in the study	
[x]	[ ]	ChIP-seq
[ ]	[x]	Flow cytometry
[x]	[ ]	MRI-based neuroimaging

Antibodies

Antibodies used	Mouse anti-PFKP Santa Cruz Biotechnology, 1:500 (WB), Cat#sc-514824;<br>Rabbit anti-PKM2 Cell Signaling Technology, 1:1000 (WB), Cat#4053;<br>Mouse anti-PKM2 Santa Cruz Biotechnology, 1:50 (IF), Cat#sc-365684;<br>Mouse anti-ENO1 Santa Cruz Biotechnology, 1:500 (WB), Cat#sc-271384;<br>Rabbit anti-PGK1 Abcam, 1:1000 (WB), Cat#ab199438;

	Rabbit anti-GPI Abcam, 1:1000 (WB), Cat#ab167394;<br>Mouse anti-p53 Santa Cruz Biotechnology,1:500 (WB), Cat#sc-126;<br>Mouse anti-p21 Santa Cruz Biotechnology,1:500 (WB), Cat#sc-6246;<br>Rabbit anti-p21 Cell Signaling Technology,1:1000 (WB), Cat#2947;<br>Rabbit anti-p16 Zen-BIO,1:1000 (WB), Cat#380963;<br>Mouse anti-p16 Santa Cruz Biotechnology,1:500 (WB), Cat#sc-1661;<br>Rabbit anti-LC3, Sigma-Aldrich,1:10000 (WB), Cat#L8918;<br>Rabbit anti-LAMP2a Zen bio,1:1000 (WB), Cat#R24835;<br>Rabbit anti-CLAR Zen bio,1:1000 (WB), Cat#380959;<br>Rabbit anti-GAPDH Cell Signaling Technology,1:5000 (WB), Cat#5174;<br>Rabbit anti-PEX19 Zen bio1:1000 (WB), Cat#389339<br>Rabbit anti-p70S6K Cell Signaling Technology,1:1000 (WB), Cat#9202;<br>Mouse anti-β-actin Santa Cruz Biotechnology,1:2000 (WB), Cat#sc-8432;<br>Mouse anti-PCNA Zen bio,1:1000 (WB), Cat#200947-2E1;<br>Rabbit anti-VDAC Cell Signaling Technology,1:1000 (WB), Cat#4866;<br>Rabbit anti-HSPA8 HUABIO,1:1000 (WB), Cat#ET1602-33<br>Mouse anti–HK2 Abcam,1:1000 (WB), Cat#ab104836;<br>Mouse anti–HA Thermo Fisher Scientific,1:50000 (WB), Cat#26183;<br>Donkey anti-rabbit Alexa Fluor 488 Thermo Fisher Scientific,1:1000 (IF), Cat#A-21206;<br>Donkey anti-mouse Alexa Fluor 594 Thermo Fisher Scientific,1:1000 (IF), Cat#A-21203;<br>Anti–PHGDH Santa Cruz Biotechnology,1:500 (WB), Cat#sc-100317;<br>Anti–MTHFD1 Santa Cruz Biotechnology1:500 (WB), Cat#sc-271412;<br>Anti-LAMP-2 BioLegend,1:2000 (WB), Cat#354301.
Validation	Antibodies were validated by manufactures or validated in previous studies. Statements on antibodies validation are present on the manufacturer's websites along with relevant references.

Eukaryotic cell lines

Policy information about cell lines and Sex and Gender in Research

Cell line source(s)	HEK 293T, MCF-7 were obtained from American Type Culture Collection (ATCC). HeLa were a kind gift from Qing Chang (Peking university), Zhengfan Jiang (Peking university) respectively. Fibroblasts 2BS were all from Zebin Mao (Peking university). Fibroblasts IMR-90 were all from Tanjun Tong (Peking university).
Authentication	No authentication was performed.
Mycoplasma contamination	All cell lines were tested negative for mycoplasma contamination.
Commonly misidentified lines<br>(See ICLAC register)	No commonly misidentified cell lines were used.

Animals and other research organisms

Policy information about studies involving animals; ARRIVE guidelines recommended for reporting animal research, and Sex and Gender in Research

Laboratory animals	Female BALB/c (8 weeks old); male C57BL/6J (8 weeks old); male C57BL/6J (18months old)
Wild animals	Female BALB/c (8 weeks old) and male C57BL/6J (8 weeks old) were obtained from the Department of Laboratory Animal Science of Peking University Health Science Center, Beijing. The 18-month-old mice were purchased from Aniphe Biolab.
Reporting on sex	The finding dose not apply to only one sex. We used the same sex in a single experiment.
Field-collected samples	N/A
Ethics oversight	Animal experiments were proven by the Institutional Animal Care and Use Committee of Peking University Health Science Center.

Note that full information on the approval of the study protocol must also be provided in the manuscript.

Plants

Seed stocks: N/A

Novel plant genotypes: N/A

Authentication: N/A

Flow Cytometry

Plots

Confirm that:

☒ The axis labels state the marker and fluorochrome used (e.g. CD4-FITC).

☒ The axis scales are clearly visible. Include numbers along axes only for bottom left plot of group (a 'group' is an analysis of identical markers).

☒ All plots are contour plots with outliers or pseudocolor plots.

☒ A numerical value for number of cells or percentage (with statistics) is provided.

Methodology

Sample preparation: HeLa cells with stable expression of vector, sfcherry or sfcherry-PKM2 were collected and lysed with lysis buffer (50 mM Tris-HCl pH 7.9, 137 mM NaCl, 1% Triton X-100, 0.2% Sarkosyl, 1 mM Na3VO4, and 10% glycerol) for 40 minutes followed by ultrasonication (30% power; 1s on, 3s off) for 20s. Then, the lysate was centrifuged at 1,000 x g, 4℃ for 3 minutes. The supernatant was transferred to a new tube followed by centrifuging at 5,000 x g, 4℃ for 10minutes. Next, discarding the supernatant and washing the pellets with lysis buffer by gently pipetting followed by centrifuging at 5, 000 x g, 4℃ for 10minutes followed by washing the pellets and centrifuging again. Finally, resuspending the pellets with lysis buffer and sorting with flow cytometry (Symphony S6).

Instrument: BD FACSymphony™ S6

Software: FlowJo v10.6.2

Cell population abundance: The abundance of PKM2 aggregates was 12%.Vector and sfcherry group were negative control.

Gating strategy: HeLa cells stably expressed with vector, sfcherry, sfcherry-PKM2 were collected and lysed followed by FACS to isolate PKM2 aggregates. Gating strategy was based on the intensity of sfcherry and SSC-A parameters of Cytometric beads.

☒ Tick this box to confirm that a figure exemplifying the gating strategy is provided in the Supplementary Information.
